# Survival of *Lawsonia intracellularis* in porcine peripheral blood monocyte-derived macrophages

**DOI:** 10.1371/journal.pone.0236887

**Published:** 2020-07-31

**Authors:** Carlos Eduardo Real Pereira, Talita Pilar Resende, Aníbal G. Armién, Ricardo Pereira Laub, Fabio Augusto Vannucci, Renato Lima Santos, Connie Jane Gebhart, Roberto Mauricio Carvalho Guedes

**Affiliations:** 1 Department of Veterinary Clinic and Surgery, Veterinary School, Universidade Federal de Minas Gerais, Belo Horizonte, Minas Gerais, Brazil; 2 Department of Veterinary and Biomedical Sciences, College of Veterinary Medicine, University of Minnesota, St. Paul, Minnesota, United States of America; 3 Department of Population Medicine, College of Veterinary Medicine, University of Minnesota, St. Paul, Minnesota, United States of America; 4 Ultrastructural Pathology Unit, Veterinary Diagnostic Laboratory, College of Veterinary Medicine, University of Minnesota, St. Paul, Minnesota, United States of America; Universidad de Costa Rica, COSTA RICA

## Abstract

*Lawsonia intracellularis*, an obligately intracellular enteric bacterium, infects intestinal epithelial cells, but may also be found within macrophages in the intestinal lamina propria of affected pigs. Macrophages play an important role in host defense against infectious agents, but the role of this cell in *L*. *intracellularis* infection is not well understood. The aim of this study was to evaluate the permissibility of macrophages to *L*. *intracellularis* infection *in vitro*. Pure culture of *L*. *intracellularis* was added to swine peripheral blood monocyte-derived macrophages. Viability of intracytoplasmic *L*. *intracellularis* was evaluated at different time points by transmission electron microscopy (TEM). Potential replication of *L*. *intracellularis* in macrophages was also evaluated by qPCR. By TEM, phagocytosis *L*. *intracellularis* within of phagolysosomes were observed 1-hour post-infection (hpi) and bacterial structures in binary fission at 48 hpi. The number of intracellular bacteria was determined at 1, 4, 24, 48, and 72 hpi by qPCR in infected macrophages and compared to the number of intracellular bacteria from culture in McCoy cells. In both cell lines, the amount of *L*. *intracellularis* was decreased at 4 hpiand increased at 24 hpi. The number of intracellular bacteria continued to increase in McCoy cells over time. This is the first study showing interaction, survival and propagation of *L*. *intracellularis* in macrophages. These findings are critical to establish an experimental model for future studies of the pathogenesis of porcine proliferative enteropathy and the potential persistence of *L*. *intracellularis* in macrophages during chronic infections.

## Introduction

Macrophages play an important role in innate and adaptive immune responses against invading pathogens. They contribute to the elimination of microorganisms and are capable of presenting antigens to T cells. In addition, macrophages may serve as an escape route for some bacterial species, as intracellular bacteria may remain viable and potentially proliferate and/or disseminate in the host [1, 2].

*Lawsonia intracellularis* is a microaerophilic, obligately intracellular bacterium that causes porcine proliferative enteropathy (PPE) [3]. PPE is responsible for economic losses worldwide in the pig industry, not only due to diarrhea, but also due to reduced weight gain, treatment and control costs of the disease [4]. Other animal species are also susceptible to *L*. *intracellularis* infection. In horses, proliferative enteropathy is considered an emerging disease [5]. In non-human primates, there are reports of outbreaks of the hemorrhagic form of proliferative enteropathy, causing sudden death in affected animals [6, 7].

The pathogenesis of *L*. *intracellularis* is still poorly understood. Infection occurs via the fecal-oral route, and in the gastrointestinal tract the microorganism overcomes the hostile environment of the stomach by enzymatic and protein mechanisms [8]. Arriving at the more aboral region of the small intestine (ileum), the bacterium uses a single polar flagellum [4] to overcome the intestinal mucus barrier, allowing an intimate contact between *L*. *intracellularis* and the intestinal epithelial cells. Then, *L*. *intracellularis* gains entry into the host cell cytoplasm by endocytosis. In the intracellular environment, *L*. *intracellularis* is able to proliferate by binary fission [9, 10].

The most likely theory for infection and *in vivo* propagation of *L*. *intracellularis* is that the bacteria infect only cells with high mitogenic potential, such as intestinal crypt cells (immature and undifferentiated cells) and is dependent on host cell mitosis for propagation [11–13]. It is believed that *in vivo* propagation of *L*. *intracellularis* occurs from enterocyte to enterocyte, by exfoliation and rupture of cells with high bacterial load, release of these microorganisms into the intestinal lumen, and infection of adjacent immature enterocytes [14–16]. Contradictory and inconclusive observations have suggested a potential role of macrophages in the spread of *L*. *intracellularis* infection. Johnson and Jacoby and Umemura et al. have demonstrated, through electron microscopy, partially degraded bacteria within macrophages of the lamina propria in tissues from infected animals [16, 17]. On the other hand, Boutrup et al., using fluorescence *in situ* hybridization (FISH), observed the presence of viable *L*. *intracellularis* within the mononuclear cells in the lamina propria [4]. FISH uses a fluorochrome-conjugated oligonucleotide probe that targets the 16s gene of ribosomal RNA (rRNA). The use of this target is justified by the fact that there is hybridization only in metabolically-active microorganisms at the moment of tissue fixation. Therefore, only viable organisms would present intact rRNA, and hence, be permissive to hybridize the probe. In addition, since all eukaryotic cells require translational ribosomes, ribosomes are present in large quantities in all metabolically-active cells [18].

To our knowledge, there is no previously reported studies that have unambiguously revealed the role of macrophages in *L*. *intracellularis* infection. To better understand this interaction, the present work aimed to evaluate the survival of *L*. *intracellularis* inside macrophages derived from peripheral blood mononuclear cells (PBMC) of pigs.

## Materials and methods

### Ethics statement

The study was conducted in accordance with all applicable legislation, and the experimental protocol has been approved by the Ethics in Animal Use Committee at the Universidade Federal de Minas Gerais (CEUA/UFMG protocol number 249/2015).

### Pure culture propagation

*L*. *intracellularis* strain PHE/MN1-00 (ATCC PTA-3457), previously isolated from a pig with the hemorrhagic form of PPE, was used in passages ranging from 12 to 14. Pure culture of the bacterium was thawed and grown in McCoy cell culture, using Dulbecco´s modified Eagle medium (DMEM—Gibco, Carlsbad, US) supplemented with 1% L-glutamine (Gibco Invitrogen Corporation, 25030–081) and 7% fetal bovine serum (FBS, Sigma Chemical). The infected cell cultures were incubated in bags with an atmosphere of approximately, 6% oxygen and 8% carbon dioxide [19], for three consecutive passages to allow the bacterium to recover from the freezing condition. Subsequently, the culture supernatant was filtered through 0.8 μm filters (Merck Millipore, Darmstadt, Germany) to remove the McCoy cells prior to infecting the PBMC culture.

### Isolation, culture and infection of macrophages

For PBMC isolation, 60 mL of blood were collected from a healthy growing pig from the swine herd located the Saint Paul campus of University of Minnesota, using heparin to prevent clotting. Then, the non-coagulated blood was diluted in DMEM medium (Gibco, Carlsbad, US) to a ratio of 1:1. The blood/DMEM mixture was diluted in Histopaque (Sigma) at the ratio of 1 Histopaque:2 blood/DMEM and centrifuged at 1200 g for 40 min at 18°C. The layer containing mononuclear cells was collected using a sterile Pasteur pipette and then centrifuged at 1200 g for 15 min at 4°C. The pellet was resuspended in lysis buffer for 5 minutes at room temperature, then added to DMEM (10 mL), and centrifuged at 1200 g for 15 min at 4°C. The pellet was finally resuspended in 10 mL RPMI-1640 medium supplemented with 10% fetal bovine serum, L-glutamine (200 mM), sodium bicarbonate (7.5% w/v), non-essential amino acids (10 mM), pyruvate (10 mM), penicillin (50 IU/mL) and streptomycin (50 μL/100 mL). The number of cells in the suspension was counted in a Neubauer’s chamber, transferred to Teflon flasks (Nalge Nunc, Rochester, US), and incubated at 37°C with 5% CO_2_. The medium was changed to remove dead and non-adherent cells and to remove the antibiotic at 24 hours after incubation. The culture was maintained in the same conditions for 10 days with medium changes every 3 days [20]. After 10 days of culture, the macrophage cells were seeded in a 6 or 24-well plate for ultrastructural and quantitative evaluation of the interaction between the macrophages and *L*. *intracellularis*.

### Transmission electron microscopy

Electronic microscopy was used to elucidate: a) whether viable *L*. *intracellularis* organisms were internalized in macrophage, b) whether bacterial organisms could escape the phagocytic digestion and c) whether bacterial organisms could be observed free of membrane-bound vacuoles in the cell cytoplasm as they are in intestinal epithelial cells. Wells containing macrophages were infected with MOI (multiplicity of infection) of approximately 10 bacteria to 1 macrophage, by using q PCR (described below) to determine the concentration of *L*. *intracellularis* in the inoculum. Then, macrophages, were fixed and evaluated at two different time points post-infection as follows: 1-hour post infection (early infection) and 48 hours post-infection (late infection). As a negative control, uninoculated macrophages were maintained in similar conditions to the inoculated group and examined with electronic microscopy.

Inoculated macrophage cultures were fixed in a solution containing 3% glutaraldehyde in 0.1 M sodium cacodylate buffer, pH 7.2, for 4 hours at 4°C. Then the macrophages were resuspended using a cell scraper, transferred to microtubes and centrifuged for 10 minutes at 1000 g at room temperature. Infected macrophages were post-fixed with 1% osmium tetroxide and 0.8% potassium ferrocyanide in 0.1 M sodium cacodylate buffer (all reagents from Electron Microscopy Sciences, Hatfield, PA, USA). After three washes in distilled water, samples were dehydrated using a 25%–100% ethyl alcohol gradient. Samples were then infiltrated with 2:1 ethanol: EMbed 812 resin (Electron Microscopy Sciences, Hatfield, PA, USA) for 1 hour and subsequently transferred to a 1:2 ethanol: EMbed 812 resin mixture for 1 hour. Macrophages were further infiltrated with 100% resin and were embedded and incubated at 58°C for 24 hours to polymerize the resin. Embedded samples were trimmed and sectioned on a Leica UC6 ultramicrotome (Leica Microsystems, Vienna, Austria). Thin sections (60–70 nm) were obtained and collected on a 200 mesh Nickle grid (Electron Microscopy Sciences, Hatfield, PA, USA). Grids were contrasted with 5% uranyl acetate for 20 minutes and Santos’ lead citrate for 6 minutes. These samples were visualized using a JEOL 1400 transmission electron microscope (JEOL LTD, Tokyo, Japan). Images were obtained using an AMT Capture Engine Version 7.00 camera and software (*Advanced Microscopy Techniques Corp*. Woburn, MA, USA).

### Quantification by qPCR

After 1 (T0), 6 (T1), 24 (T2), 48 (T3), and 72 (T4) hours of culture, cells were washed three times with PBS, lysed and processed for bacterial DNA extraction using DNeasy Blood & Tissue Kit^®^, according to with instructions from the manufacturer (Qiagen, Valencia, CA, USA). The extracted DNA was quantified by the qPCR technique [21]. Additionally, the inoculum was quantified in the same way. As a positive control, McCoy cells infected with *L*. *intracellularis* were used to confirm the extraction and to create a bacterial growth curve.

### Statistical analysis

Two-way ANOVA followed by Bonferroni’s multiple comparisons test was used to compare bacterial growth between macrophages and McCoy cells. A *p < 0*.*05* was considered statistically significant.

## Results

### Transmission electron microscopy

**Fig 1:**
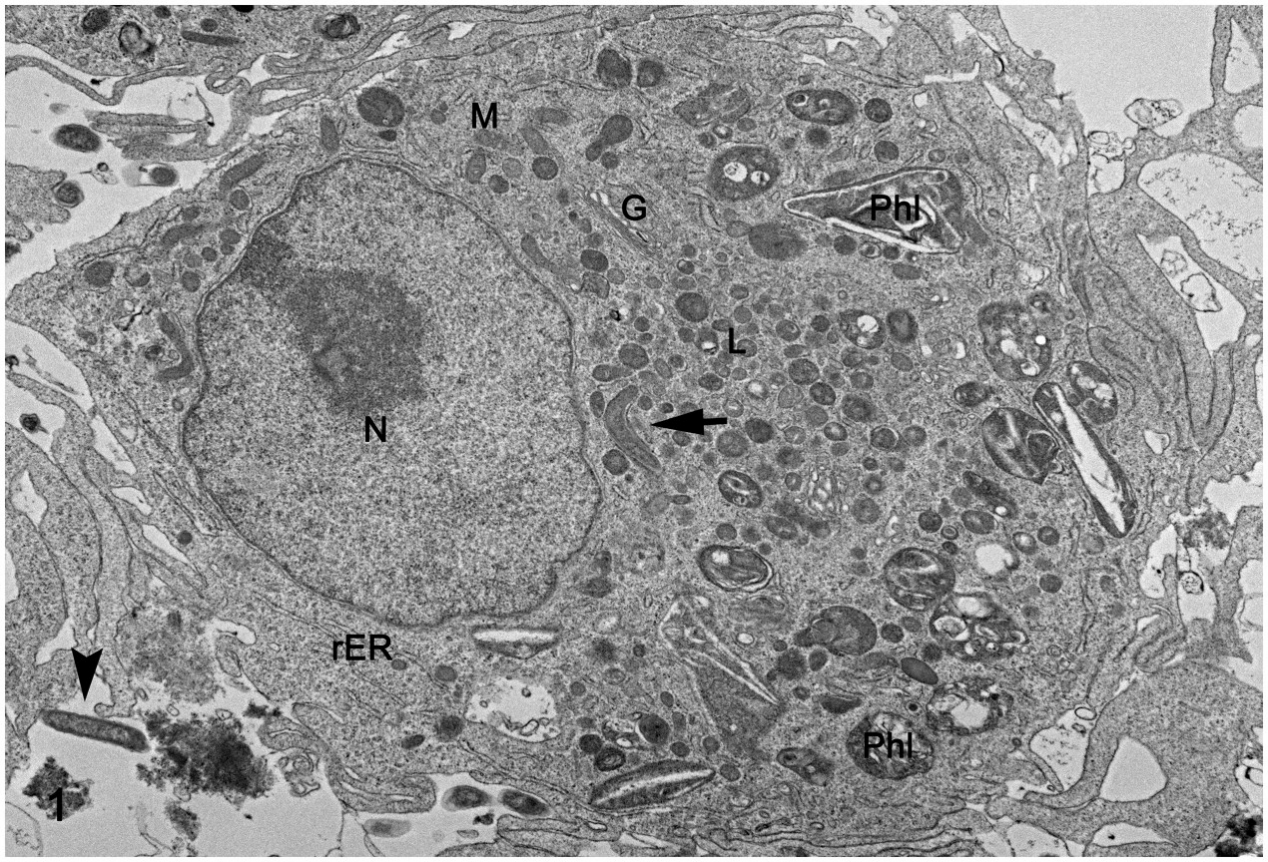
Monocyte-derived macrophage infected with *Lawsonia intracellularis*. Transmission electron microscopy. At one-hour post infection, *L*. *intracellularis* was observed in the proximity of the macrophage membrane (arrowhead) and free within the macrophage cytoplasm (black arrow). G: Golgi apparatus; L: lysosome; M: mitochondria; N: nucleus; Phl: phagolysosome; rER: rough endoplasmic reticulum.

**Fig 2:**
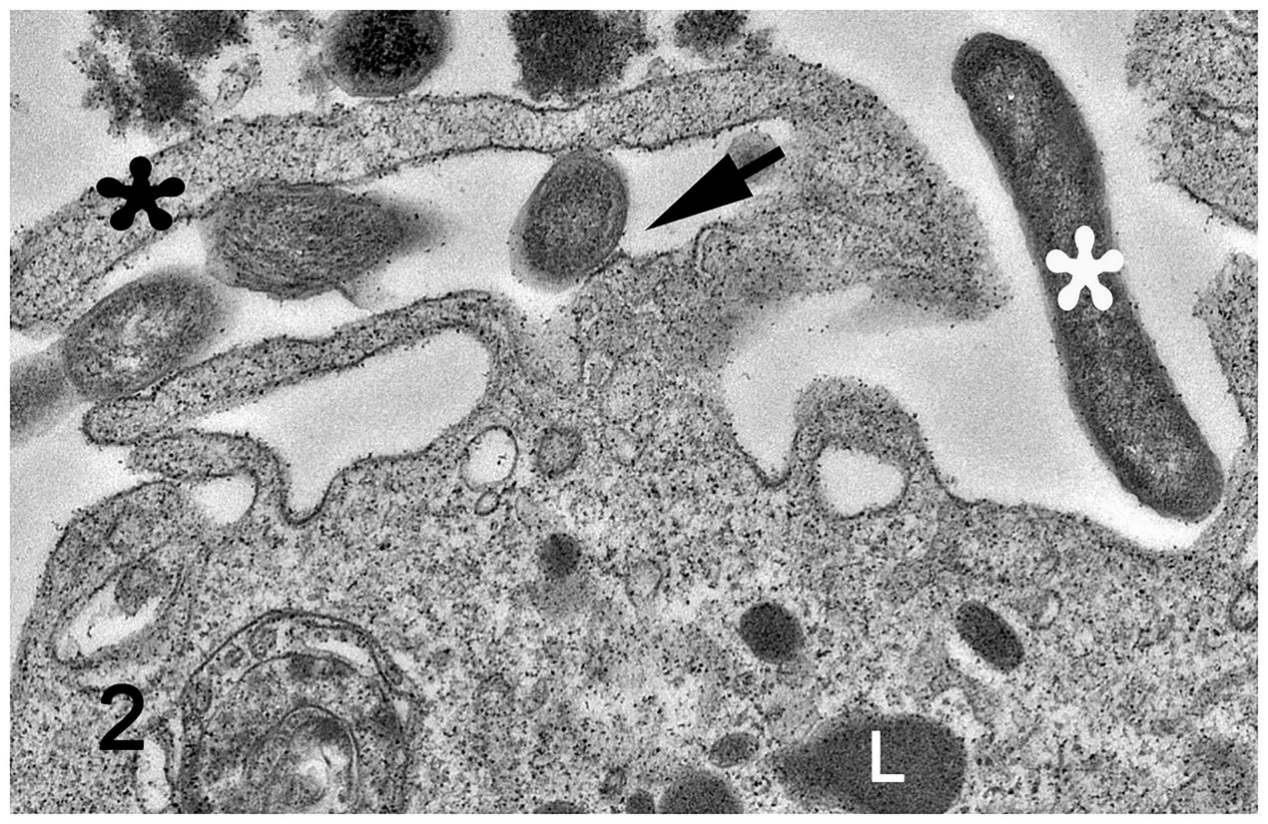
Monocyte-derived macrophage infected with *Lawsonia intracellularis*. Transmission electron microscopy. Cross-sectioned *L*. *intracellularis* was attached to the macrophage surface, where an electrodense interface between bacteria and macrophage plasmamembrane is observed (black arrow), adjacent to formation of a phyllopod (black asterisk) at one-hour post infection. *L*. *intracellularis* in longitudinal section (white asterisk) is also observed in the proximity of the macrophage surface. L: lysosome.

**Fig 3:**
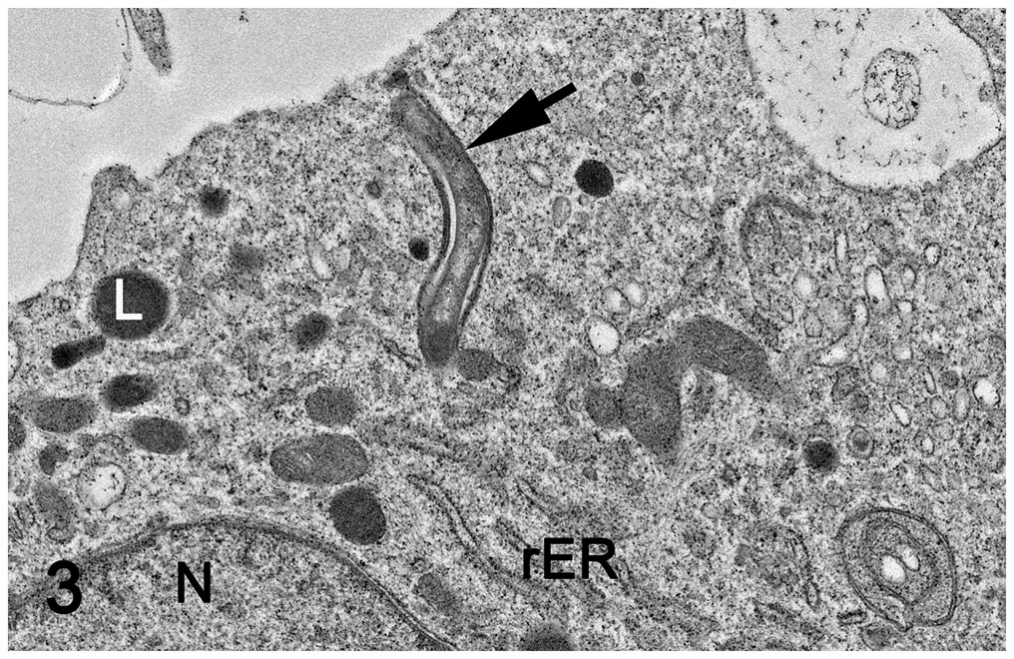
Monocyte-derived macrophage infected with *L*. *intracellularis*. Transmission electron microscopy. *L*. *intracellularis* organism was observed within phagocytic vesicle (black arrow), adjacent to the macrophage membrane. L: lysosome; N: nucleus; rER: rough endoplasmic reticulum.

**Fig 4:**
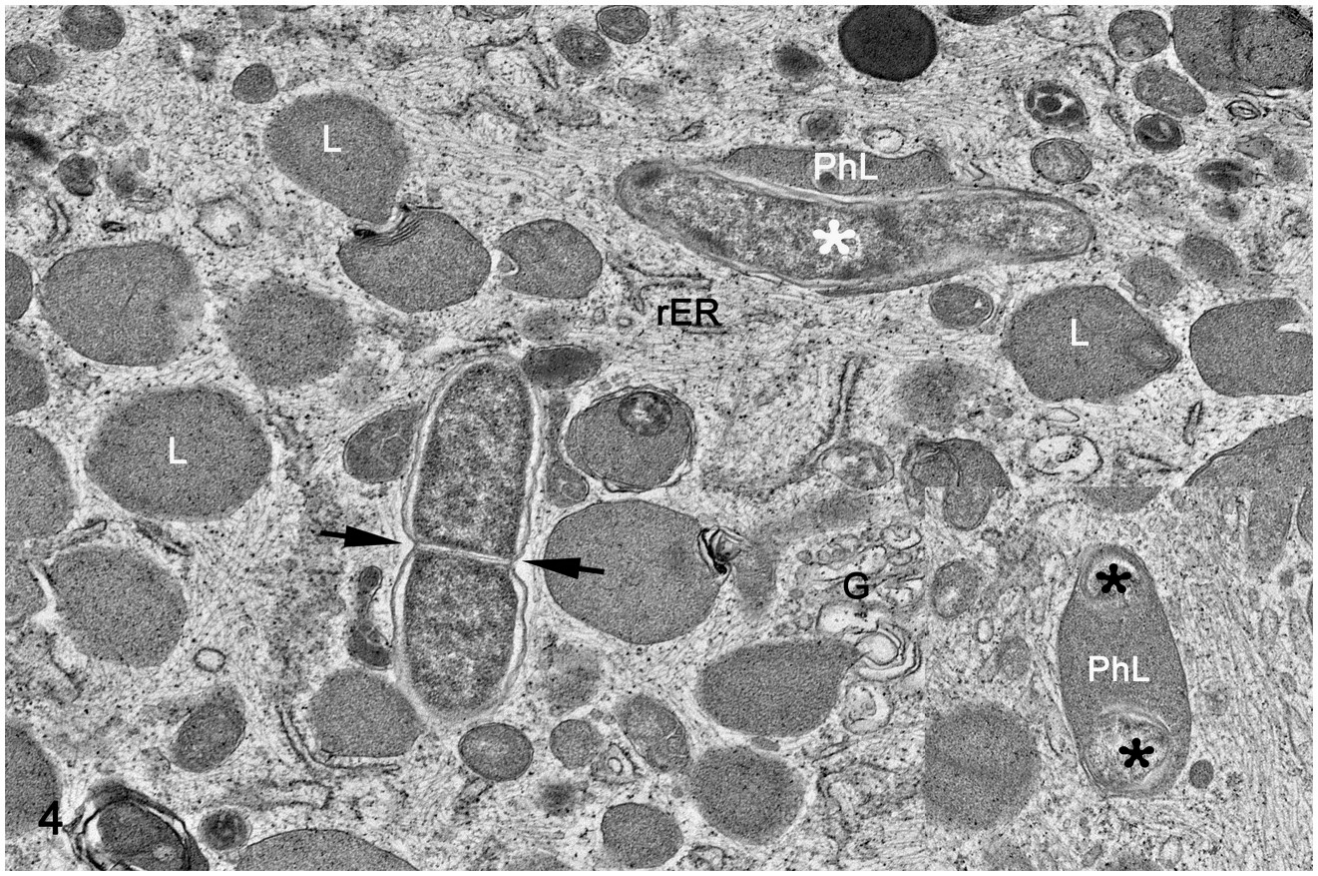
Monocyte-derived macrophage infected with *Lawsonia intracellularis*. Transmission electron microscopy. At 48 hours post infection, a sagittal section of a viable *L*. *intracellularis* organisms (white asterisks) is present within a phagolysosome (PhL). Free within the cytoplasm is a *L*. *intracellularis* organism in binary fission; black arrows show a formed wall separating the new two bacteria. G: Golgi apparatus; L: lysosome; rER: rough endoplasmic reticulum. Inset: cross section of viable *L*. *intracellularis* organisms (black asterisks) are present within a phagolysosome (PhL).

*L*. *intracellularis* organisms were observed in the proximity of the macrophage membrane and attached to the macrophage surface, where an electro dense interface between bacteria and macrophage plasma membrane is observed adjacent to formation of a phyllopod (Figs 1 and 2). Intact *L*. *intracellularis* organisms were also observed within phagocytic vacuoles (Fig 3), within phagolysosomes (Fig 4) and free in the macrophage cytoplasm (Fig 1). *L*. *intracellularis* organisms in binary fission were occasionally observed free in the cytoplasm (Fig 4). In addition, phagolysosome contained intact *L*. *intracellularis* and amorphous, membranous and lamellar arrays, presumably derived from an increased autophagy and mitophagy. A non-infected macrophage was used as negative control (S1 Fig). Increased autophagy and mitophagy was also observed in these cells in a lower degree compared to the *L*. *intracellularis*.

### qPCR

**Fig 5:**
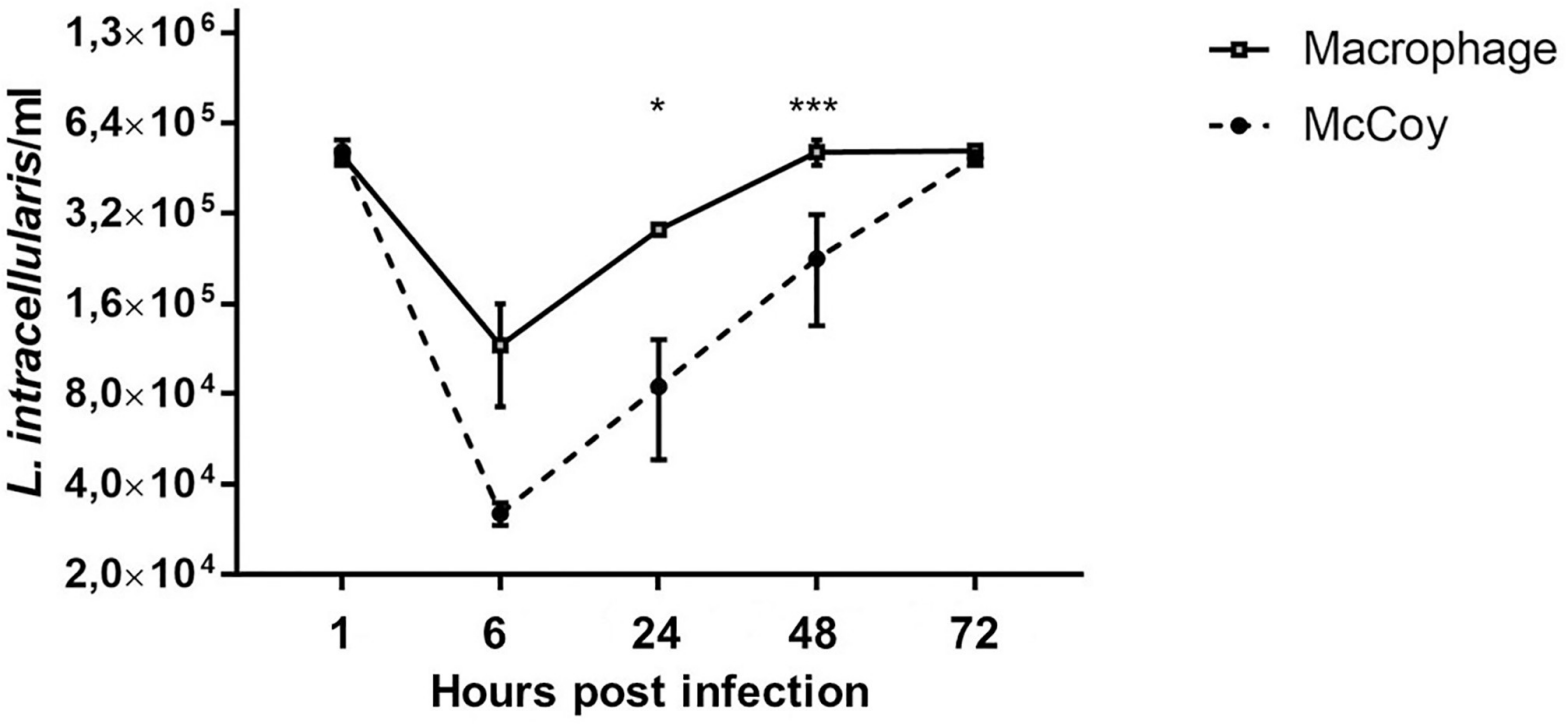
Quantification of *Lawsonia intracellularis* by qPCR. Time course of *L*. *intracellularis* mean quantification in macrophages and McCoy cells at 1, 6, 24, 48, and 72 hours post infection ± standard deviation of the mean. * *P < 0*.*05*; *** *P < 0*.*001*.

McCoy cells infected with *L*. *intracellularis* were used as a control for the bacterial propagation under conditions similar to those routinely used for *L*. *intracellularis* propagation [19]. In the McCoy cell culture, the amount of *L*. *intracellularis*, inside of the cells decreased in the first hours after infection. At later stages of infection, an increase in the bacterial numbers was observed (Fig 5). In the macrophage culture, there was less decrease in the amount of *L*. *intracellularis* in the first hours after infection, but the increase of the bacterial DNA was lower throughout the time course (Fig 5), remaining constant from T2 to T4.*L*. *intracellularis* had a higher propagation in macrophages than in McCoy cells at 24 hpi *(P < 0*.*05)* and at 48 hpi *(P< 0*.*001*).

## Discussion

*Lawsonia intracellularis*, a Gram-negative obligate intracellular bacterium is the only species in the genus and is considered to be an enteric pathogen to various animal species, including mammals and birds [22]. *L*. *intracellularis*-associated disease, named as proliferative enteropathy, has been reported since early 1910´s, but *L*. *intracellularis* has only been recognized as a bacterial species in 1993 [23]. Due the laborious process of bacterial isolation and maintenance in laboratory conditions [24], research on *L*. *intracellularis* pathogenesis is scarce. Nevertheless, based on previous [25 e 23] findings, we hypothesized that *L*. *intracellularis* can survive and propagate in macrophages [25, 26]. The present study showed intact *L*. *intracellularis* organisms within phagolysosomes in cultured macrophages, free in the macrophage cytoplasm, and in binary fission. These results indicate that *L*. *intracellularis* has the ability to survive and propagate inside macrophages. However, despite the capacity of *L*. *intracellularis* to survive within the macrophages as shown in the present study, only fortuitous cases of *L*. *intracellularis* infection are associated to extra intestinal lesions [25, 26]. These rare cases of extra-intestinal lesions associated with *L*. *intracellularis* indicate that the survival of *L*. *intracellularis* in macrophages is not related to the dissemination of the bacterium to extra intestinal tissues, as already described for other intestinal gram-negative bacteria [27, 28]. Direct methodologies to confirm the replication and to determine the molecular mechanisms used by *L*. *intracellularis* to survive intracellularly whether in epithelial cell or macrophages are as still required.

The survival of bacteria in phagolysosomal compartments has been observed in other species, such as *Rhodococcus equi* and *Coxiella burnetii* [8, 29]. *R*. *equi* survive and proliferate within intracellular environment by blocking the acidification of the phagolysosome [30]. On another hand, *C*. *burnetii* delays the fusion process between the phagosome and the lysosome [29], which may prompt the synthesis of protective molecules in the hostile environment of the phagolysosomes [31]. Binary fission of intracellular bacterial organisms is well documented for several bacteria [32]. However, only some bacterial species, such as *Listeria monocytogenes* [33], *Franscisella tularensis* [32], and *Edwardsiella tarda* [30], are known to replicate freely in the cytoplasm of host cells. The knowledge regards the intracellular traffic of these bacteria might serve to design future studies of *L*. *intracellularis* pathogenesis.

*L*. *intracellularis* survival within the macrophages may also be related to the persistence of the microorganisms in the host, which results in the chronic presentation of the disease. Therefore, the elimination of *L*. *intracellularis* for long periods, as previously demonstrated by Guedes and Gebhart and Stege et al, may be a source of dissemination of the pathogen in the herd [34, 35].

To assess the bacterial numbers in the macrophage cultures over time, qPCR was performed. This technique detects DNA sequences with good sensitivity and specificity but lacks the ability to differentiate metabolically active and inactive microorganisms. In addition, the chronological evaluation was performed by using samples from different wells, which can result in numerical variations from well to well. Nevertheless, from the results obtained, we can hypothesize that *L*. *intracellularis* has the capacity to proliferate at low levels within the macrophages, but other methods should be used in future studies to confirm this hypothesis. Our findings do not corroborate Lawson et al. [36] findings, who have indicated that *L*. *intracellularis* needs mitotically active cells for its proliferation. *L*. *intracellularis* is a unique bacterial species, with several peculiarities obligately intracellular microorganism, requires specific microaerophilic atmosphere that make it difficult to grow and manipulate under laboratory conditions. Therefore, evaluation of *L*. *intracellularis* survival and its quantification inside the cells is not possible using conventional assays used for studying other bacterial species [1, 37]. The present study is a proof-of-concept of the *L*. *intracellularis* potential to survive and to replicate within macrophages, therefore, it represents the first step towards the understanding of the persistence of the organism in chronically-infected animals.

In conclusion, our results showed that *L*. *intracellularis* is able to internalize and survive within phagolysosomal environment of swine macrophages. In addition, this is the first study to report the binary fission of *L*. *intracellularis* free in the cytoplasm of swine macrophages. These findings are critical to establish an experimental model for future studies the pathogenesis of porcine proliferative enteropathy and the potential persistence of *L*. *intracellularis* in macrophages during chronic infections.

## Supporting information

S1 Fig Monocyte-derived macrophage, negative control. L: lysosome; N: nucleus; rER: rough endoplasmic reticulum.
(TIF)
