# Supplementary figures and images for: Survival of Lawsonia intracellularis in porcine peripheral blood monocyte-derived macrophages
Source: PLoS One. 2020 Jul 31;15(7):e0236887. doi: 10.1371/journal.pone.0236887 (PMC7394435; doi:10.1371/journal.pone.0236887)

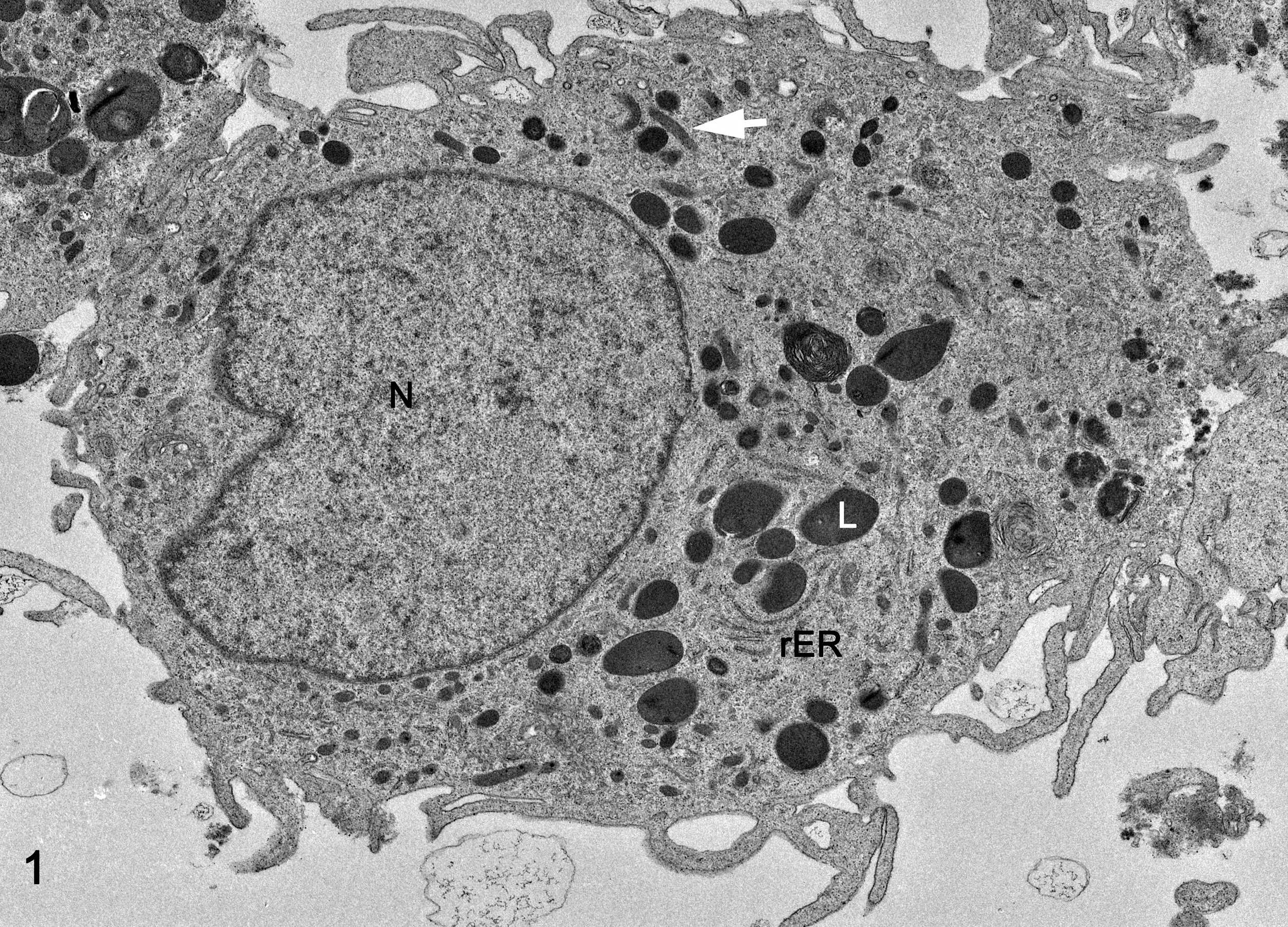

Supplement: S1 Fig — L: lysosome; N: nucleus; rER: rough endoplasmic reticulum. (TIF) [file pone.0236887.s001.tif]
